# The expression of heterologous MAM-7 in *Lactobacillus rhamnosus* reduces its intrinsic capacity to inhibit colonization of pathogen *Vibrio parahaemolyticus* in vitro

**DOI:** 10.1186/s40659-015-0064-1

**Published:** 2016-01-07

**Authors:** Sebastian Beltran, Cristian A. Munoz-Bergmann, Ana Elola-Lopez, Javiera Quintana, Cristopher Segovia, Annette N. Trombert

**Affiliations:** Escuela de Tecnología Médica, Facultad de Medicina, Universidad Mayor, Camino La Pirámide 5750, Santiago, Huechuraba, Chile; Centro de Genómica y Bioinformática, Facultad de Ciencias, Universidad Mayor, Camino La Pirámide 5750, Santiago, Huechuraba, Chile

**Keywords:** *Lactobacillus*, *V. parahaemolyticus*, Adhesion, Recombinants probiotics

## Abstract

**Background:**

*Vibrio parahaemolyticus* (*V. parahaemolyticus*) is a Gram-negative, halophilic bacterium recognized as one of the most important foodborne pathogen. When ingested, *V. parahaemolyticus* causes a self-limiting illness (Vibriosis), characterized mainly by watery diarrhoea. Treatment is usually oral rehydration and/or antibiotics in complicated cases. Since 1996, the pathogenic and pandemic *V. parahaemolyticus* O3:K6 serotype has spread worldwide, increasing the reported number of vibriosis cases. Thus, the design of new strategies for pathogen control and illness prevention is necessary. *Lactobacillus* sp. grouped Gram positive innocuous bacteria, part of normal intestinal microbiota and usually used as oral vaccines for several diarrheic diseases. Recombinants strains of *Lactobacillus* (RL) expressing pathogen antigens can be used as part of an anti-adhesion strategy where RL block the pathogen union sites in host cells. Thus, we aimed to express MAM-7 *V. parahaemolyticus* adhesion protein in *Lactobacillus* sp. to generate an RL that prevents pathogen colonization.

**Results:**

We cloned the MAM-7 gene from *V. parahaemolyticus* RIMD 2210633 in *Lactobacillus* expression vectors. Recombinant strains (*Lactobacillus rhamnosus* pSEC-MAM7 and *L. rhamnosus* pCWA-MAM7) adhered to CaCo-2 cells and competed with the pathogen. However, the *L. rhamnosus* wild type strain showed the best capacity to inhibit pathogen colonization in vitro. In addition, LDH-assay showed that recombinant strains were cytotoxic compared with the wild type isogenic strain.

**Conclusions:**

MAM-7 expression in lactobacilli reduces the intrinsic inhibitory capacity of *L. rhamnosus* against *V. parahaemolyticus.*

## Background

*Vibrio parahaemolyticus* (*V. parahaemolyticus*) is a Gram-negative halophilic bacterium; one of the most important foodborne pathogens worldwide. Autochthonous to estuarine, marine and coastal environments, *V. parahaemolyticus* causes acute gastroenteritis by consumption of raw, undercooked and/or mishandled seafood [1]. The illness vibriosis is mainly characterized by diarrhoea. It is self-limiting and usually treated by oral rehydration. In some cases antibiotics are necessary [1]. Recently, the CDC reported an increment of 75 % in vibriosis cases in the US during 2013, compared with the period 2006–2008. During 2010–2013, the increment observed was 32 %. Considering that the CDC estimates that for every *V. parahaemolyticus* case reported, there are 142 cases not diagnosed, official data are clearly underestimated [http://www.cdc.gov/foodnet/data/trends/trends-2013-progress.html]. In Chile, according the Epidemiology Department of the Chilean Ministry of Health, during the summer of 2015, there was an increase of nearly 56 % of the number of cases in comparison with 2014 (Departamento de Epidemiología, Ministerio de Salud de Chile, 2015. http://epi.minsal.cl/). Thus, it is imperative to design new strategies for the control of this pathogen.

Pandemic *V. parahaemolyticus* O3:K6 strain was first detected in Osaka (Japan) in 1950, and since 1996 this serotype has been spread throughout India, Europe, Africa, North, Central and South America [1]. In addition to classical *V. parahaemolyticus* TDH and TRH virulence factors, O3:K6 exhibited specific genetic markers as *toxRS/New* and *orf8* [2, 3]. Additionally, *V. parahaemolyticus* O3:K6 strain RIMD 2210633, was detected for the first time in Japan in 1996, encoded for Type 3 secretion system (T3SS) and a pathogenicity island (VPaI-7) [4].

In addition, an outer membrane protein, multivalent adhesion molecule (MAM) which includes MCE (from Mammalian cell entry domains) was recently described in *V. parahaemolyticus* [5, 6]. The protein, named MAM-7, can mediate pathogen attachment to mammalian cells even in the absence of other adhesion proteins [5, 6].

Because adhesion of *V. parahaemolyticus* to host cellular membranes is a crucial step for the delivery of pathogen toxins, MAM-7 could be considered as a virulence factor [7, 8]. Thus, interference with MAM-7-mediated adhesion can prevent or treat vibriosis.

Interference with the adhesion of the pathogen is known as anti-adhesion therapy (AAT). AAT includes disruption of host pathogen receptor biogenesis; analogues to compete with these receptors or antibodies block surface epitopes required for pathogen binding [9]. The analogues of adhesion can be used purified [10] or expressed (secreted or anchored in the outer membrane) in non-pathogenic bacteria. Non-pathogenic bacteria used for this purpose are those that form part of normal microbiota (as non-pathogenic *Escherichia coli*) or beneficial bacteria (probiotics) as members of acid lactic bacteria (i.e. *Lactobacillus* sp.).

*Lactobacillus* sp. are generally recognized as safe (GRAS), and can colonize human intestinal mucosa. In addition, *Lactobacillus* strains can produce inhibitory compounds such as organic acids, hydrogen peroxide and bacteriocins that have antimicrobial properties. For example, *L. plantarum* AS1 show antibacterial activity against several enteropathogens and inhibit *V. parahaemolyticus* adhesion to the human intestinal cell line HT-29 [11]. Additionally, mice treated with *L. rhamnosus* and *L. brevis* showed a significant reduction in intestinal fluid accumulation and villi damage when animals were challenged with the pathogen [12].

Given these characteristics, *Lactobacillus* strains are good candidates for mucosal vaccines and/or therapeutic delivery vehicles. In fact, recombinant lactobacilli expressing pathogen antigens can elicit mixed Th1/Th2 immune responses; induce specific antibodies, dendritic cell maturation and production of pro-inflammatory cytokines [13]. The most common strategy to generate recombinant lactobacilli is the cloning of a determined antigen-encoded gene in expression vectors [12]. One example is the use of expression vectors pSEC and pCWA to target heterologous proteins to different secreted or cell wall destinations in *Lactococcus* and *Lactobacillus* [14–16]. pSEC harbours a transcriptional fusion between the ribosome-binding site (RBS_Usp45_) and the signal peptide sequence (SP_Usp45_) of the *usp*45 gene, allowing antigen release into the medium. In addition, pCWA encoded for *Streptococcus pyogenes* M6 protein cell wall-anchor region CWA_M6_ [14–18].

We evaluated the role of recombinant *Lactobacillus* strains containing pSEC-MAM7 and pCWA-MAM7 in the adhesion to human epithelial cell lines and their capacity to inhibit *V. parahaemolyticus* colonization in vitro.

Our results showed that *L. rhamnosus* wild type can inhibit and compete in a significantly more efficient manner than both the recombinant strains pSEC-MAM7 and pCWA-MAM7. In addition, these recombinant strains showed a significantly higher grade of cytotoxicity than the *L. rhamnosus* wild type. Thus, MAM-7 expression in lactobacilli does not improve the intrinsic inhibitory capacity of *L. rhamnosus* against *V. parahaemolyticus.*

## Results

### Recombinant *L. rhamnosus* strains

**Fig. 1:**
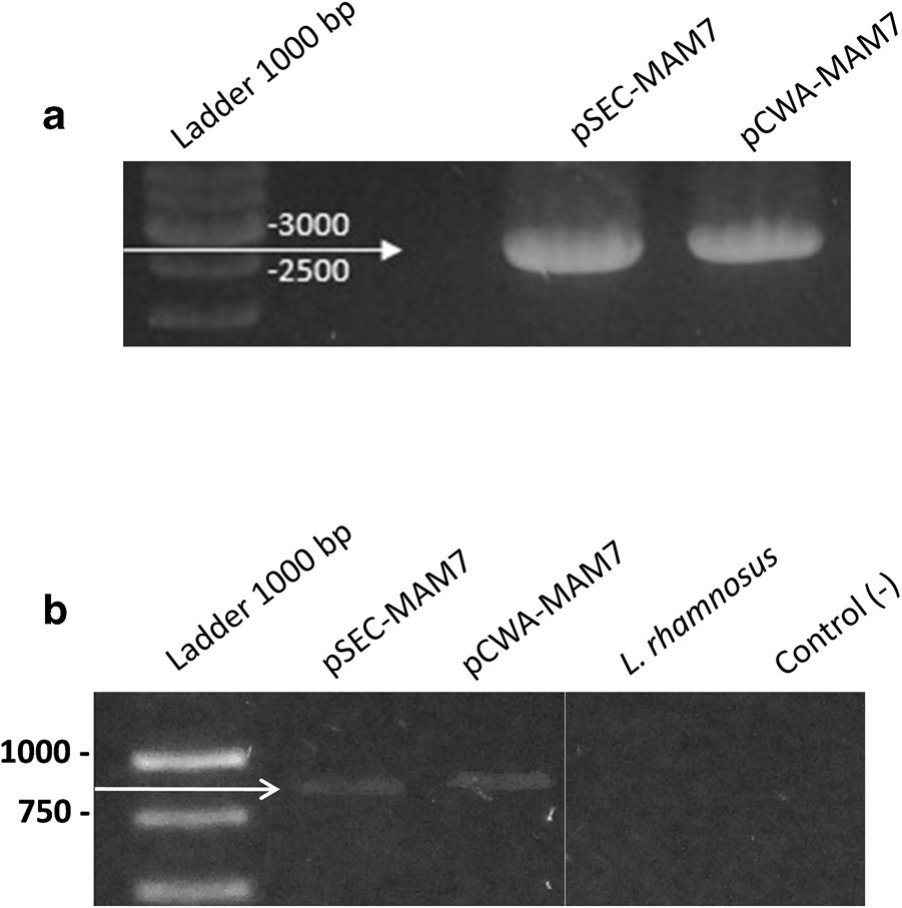
PCR and RT-PCR of MAM-7 from recombinants *L. rhamnosus* strains. **a** PCR of MAM-7 gene from *L. rhamnosus* pSEC-MAM-7 (pSEC-MAM7) and *L. rhamnosus* pCWA-MAM-7 (pCWA-MAM7) strains. **b** RT-PCR of MAM-7 transcript from *L. rhamnosus* pSEC-MAM-7 (pSEC-MAM7), *L. rhamnosus* pCWA-MAM-7 (pCWA-MAM7) and *L. rhamnosus* wild type (*L. rhamnosus)* strains

Primers were design from *V. parahaemolyticus* reported sequence RIMD 2210633 (GenBank: NC_004603). The PCR product of approx 2600 pb were cloned in pSEC and pCWA and used to transform *L. rhamnosus*. PCR was used to check the presence of MAM-7 gene in recombinant *L. rhamnosus* strains. To check if MAM-7 gene was transcribed, we performed an RT-PCR (Fig. 1). Figure 1 shows the amplicon of MAM-7 gene (Fig. 1a) and amplification of MAM-7 transcript (Fig. 1b) in both *L. rhamnosus* pSEC-MAM7 and *L. rhamnosus* pCWA-MAM7 strains.

**Fig. 2:**
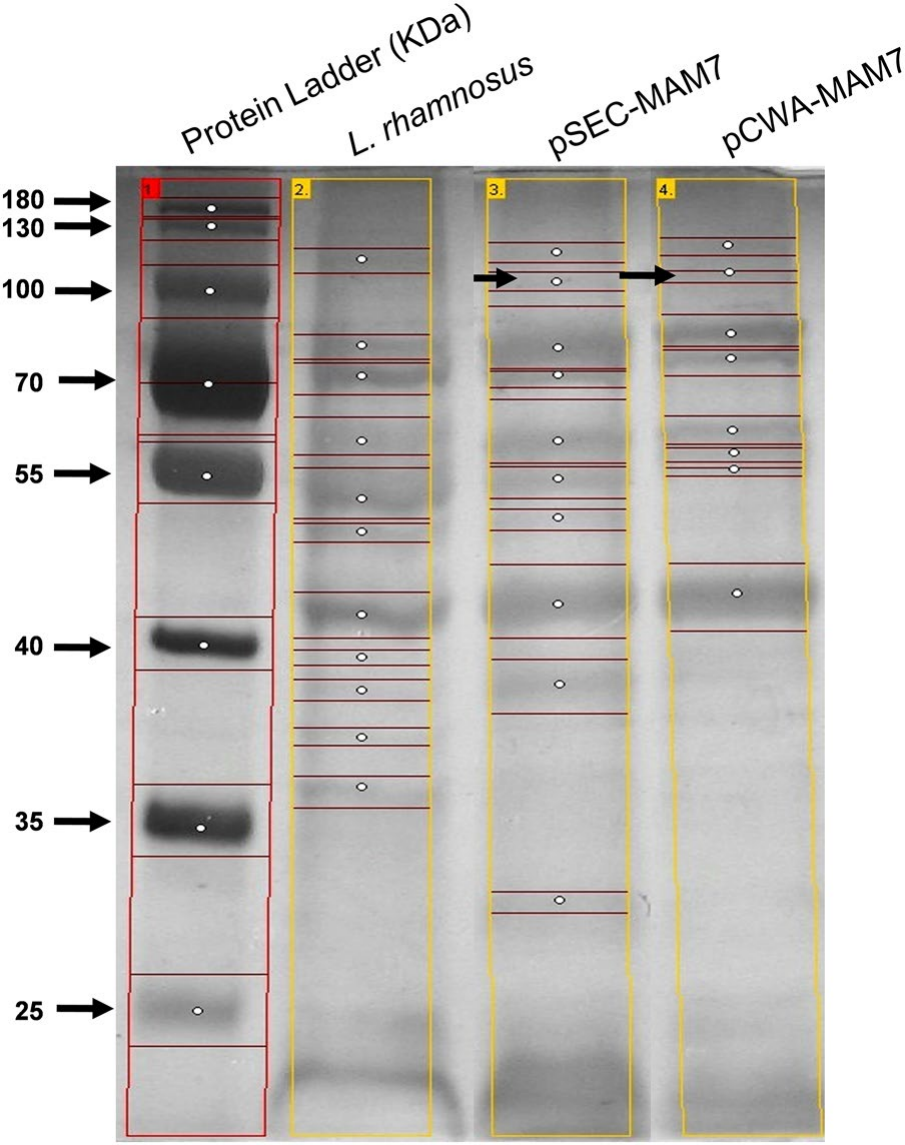
SDS-PAGE electrophoresis from recombinants *L. rhamnsus* strains. The figure shows the protein profile from *L. rhamnosus* pSEC-MAM-7 (pSEC-MAM7), *L. rhamnosus* pCWA-MAM-7 (pCWA-MAM7) and *L. rhamnosus* wild type (*L. rhamnosus*). *Black arrows* show a weakly band of approximately 100 kDa in recombinants strains. Bands of each* lane* were detected using GelAnalyzer and each* white circle* indicates a band

**Fig. 3:**
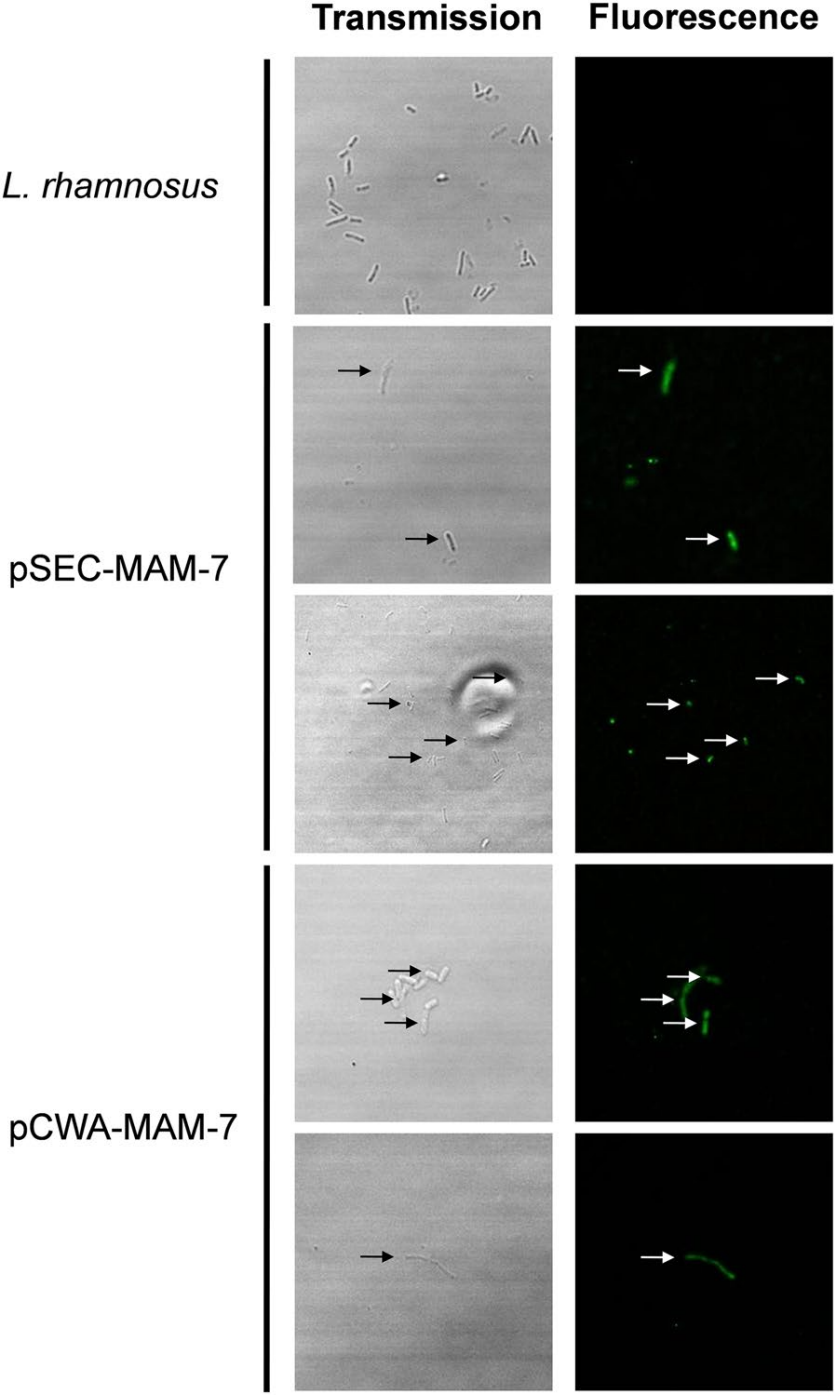
Expression of MAM-7 protein in *L. rhamnosus.* MAM-7 protein was detected using anti-*Vibrio* antibody and revealed with a secondary antibody alexa fluor^®^ conjugated. The figures shows* green fluorescence* of alexa fluor^®^ only in recombinant *L. rhamnosus* strains (*L. rhamnosus* pSEC-MAM-7 and *L. rhamnosus* pCWA-MAM-7). As a control, *L. rhamnosus* wild type was used

To assess if MAM-7 protein was expressed in both *L. rhamnosus* pSEC-MAM7 and *L. rhamnosus* pCWA-MAM7 strains, we performed an SDS-PAGE (Fig. 2) where it was expected a MAM-7 protein size of about 97 kDa. Figure 2 shows a weak band that corresponds to a protein of about 100 kDa (black arrows) suggesting the presence of MAM-7 in *L. rhamnosus* pSEC-MAM7 and *L. rhamnosus* pCWA-MAM7 strains. Thus, to check the presence of MAM-7 protein in recombinants strains, an immunodetection assay using anti-*Vibrio* (KPL) and anti-rabbit fluorescent (Molecular Probes, Thermo Fisher Scientific) antibodies was performed (Fig. 3). Figure 3 shows that while in *L. rhamnosus* wild type did not show fluorescence, both *L. rhamnosus* pSEC-MAM7 and *L. rhamnosus* pCWA-MAM7 strains showed fluorescent bacteria. The previous results strongly suggest that recombinant bacterial strains were expressing *V. parahaemolyticus* MAM-7 protein.

**Fig. 4:**
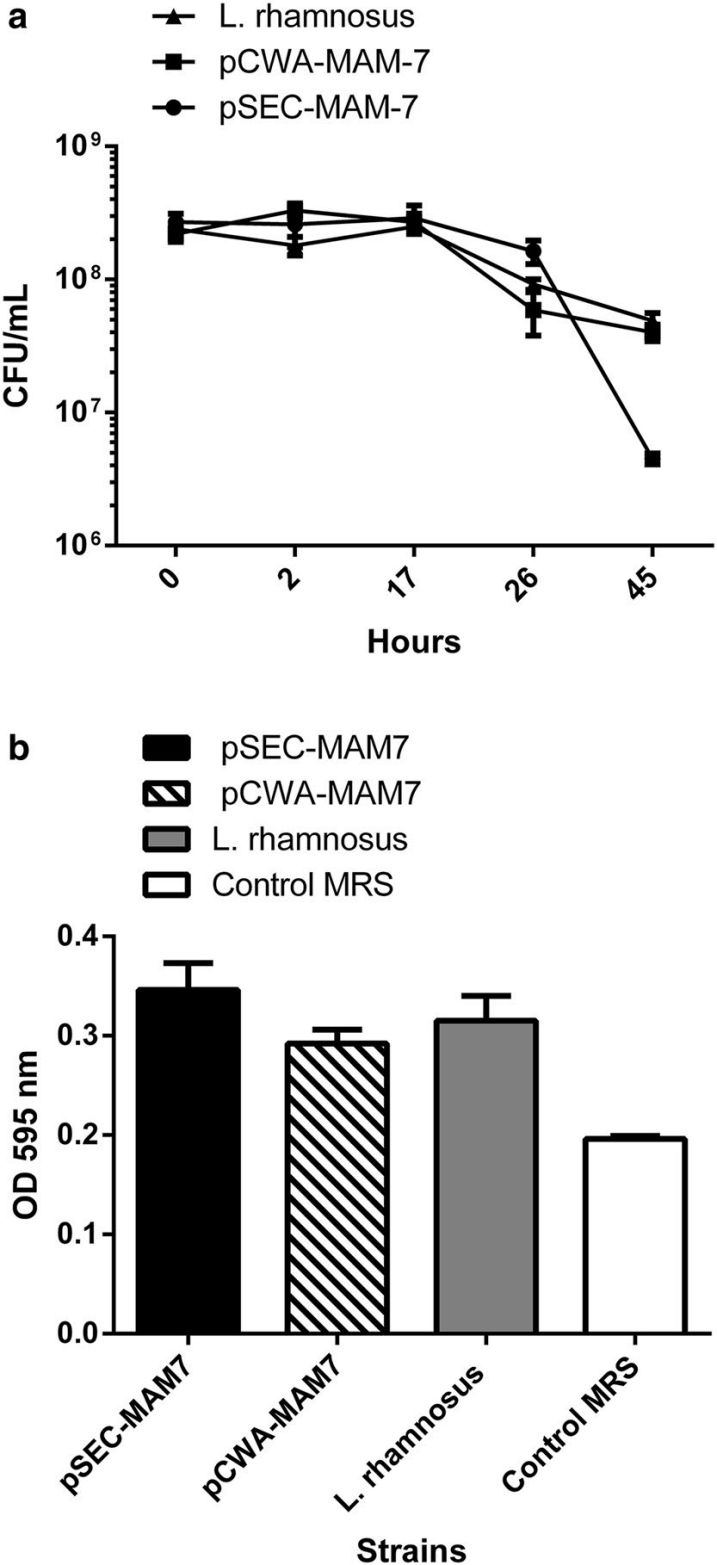
Effect of MAM-7 expression in *L. rhamnosus* in vitro growth. **a** Growth curve of *L. rhamnosus* pCWA-MAM-7 (*black squares*), *L. rhamnosus* pSEC-MAM-7 (*black circles*) recombinants strains and *L. rhamnosus* wild type strain (*black triangle*). **b** Biofilm formation of *L. rhamnosus* pSEC-MAM-7 (*black column*), *L. rhamnosus* pCWA-MAM-7 (*striped column*) recombinants strains and *L. rhamnosus* wild type strain (*grey column*). *White column* shows control with sterile MRS broth. The figure shows values expressed as the mean ± standard deviation of three full biological replicates, each time in technical triplicate

To assess if the expression of MAM-7 alters (or not) both *L. rhamnosus* pSEC-MAM7 and *L. rhamnosus* pCWA-MAM7 growth, a growth curve at 37 ℃ was performed (Fig. 4a). As shown in Fig. 4a, growth of recombinant strains was not negatively altered with the expression of MAM-7 compared with the wild type.

Adherent bacteria, such as *L. rhamnosus,* can colonize different environments as biofilms, where a self-produced extracellular matrix protects bacteria against environmental conditions [19]. Biofilm formation can be altered by the expression of the MAM-7 protein in *L. rhamnosus* recombinants strains. Therefore, we evaluated the biofilm formation capacity of *L. rhamnosus* recombinant and wild type strains using crystal violet staining protocol (Fig. 4b). Figure 4b not shows significant differences between recombinant and wild type strains. Hence, MAM-7 expression did not alter the intrinsic capacity of *L. rhamnosus* to form biofilm.

### Adhesion of recombinants *L. rhamnosus* strains to epithelial cell lines

**Fig. 5:**
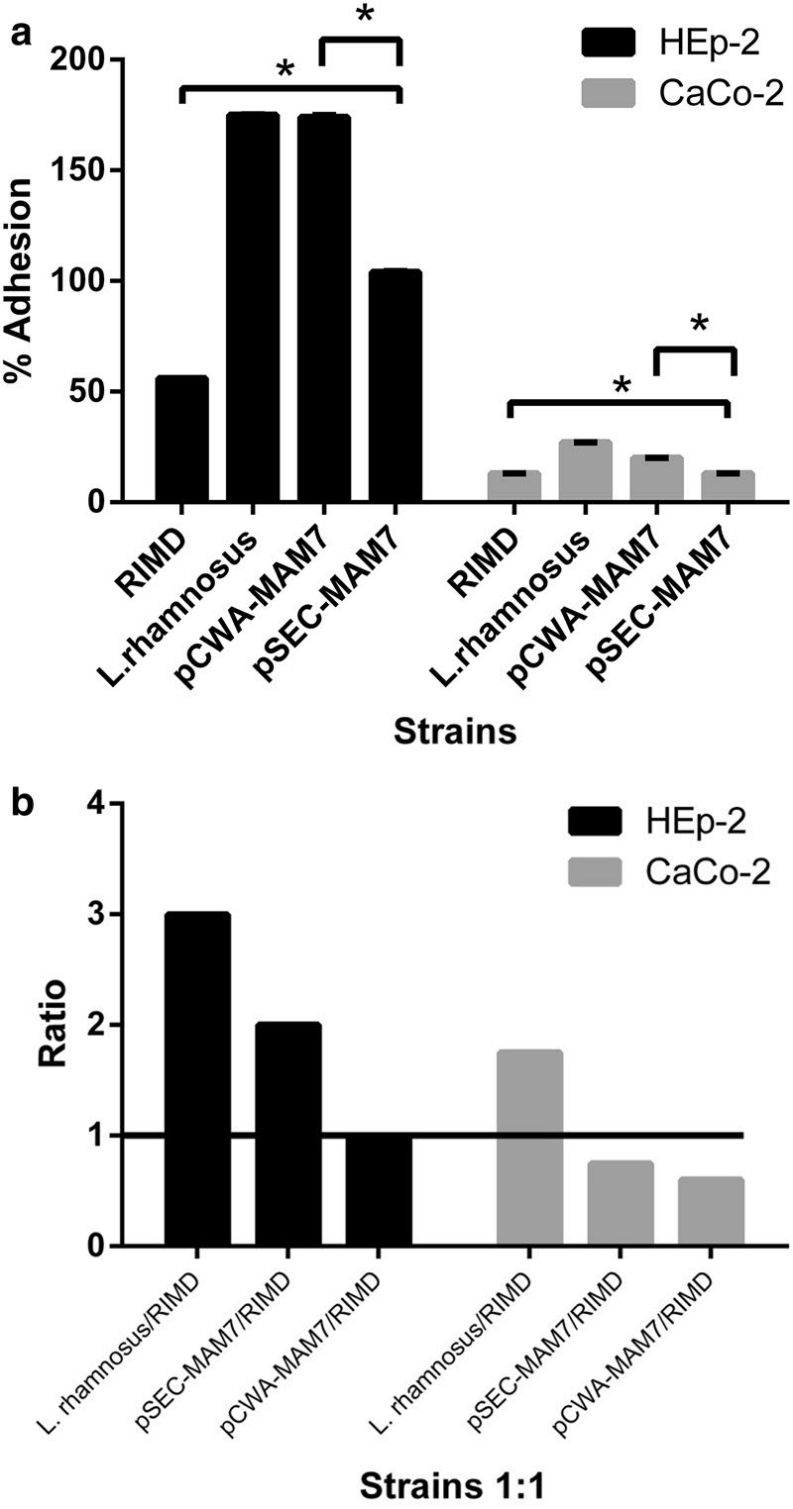
Adhesion and competition assays of *V. parahaemolyticus* and *L. rhamnosus* strains in epithelial cell lines. **a** Adhesion assay. The figure shows the adhesion percentage of *L. rhamnosus* strains and *V. parahaemolyticus* in epithelial cell line (HEp-2, *black bars*) and colon carcinoma cell line (CaCo-2, *gray bars*) monolayers. The strains used were *V. parahaemolyticus* RIMD 2210633 (RIMD), *L. rhamnosus* wild type (*L. rhamnosus*), *L. rhamnosus/*pCWA-MAM-7 (pCWA-MAM7) and *L. rhamnosus/*pSEC-MAM-7 (pSEC-MAM7). The results were expressed such as the percentage of adhesion at 3 h compared with initial inoculums of each strain at time zero. The figure shows values expressed as the mean ± standard deviation of three full biological replicates, each time in technical triplicate. *p < 0.001 (Student’s-Test). **b** Competition assay. The figure shows the results about the role of *L. rhamnosus* strains in the inhibition of *V. parahaemolyticus* from colonizing epithelial cell line (HEp-2, *black bars*) and colon carcinoma cell line (CaCo-2, *gray bars*) monolayers. The strains used were *V. parahaemolyticus* RIMD 2210633 (RIMD), *L. rhamnosus* wild type (*L. rhamnosus*), *L. rhamnosus*/pCWA-MAM-7 (pCWA-MAM7) and *L. rhamnosus*/pSEC-MAM-7 (pSEC-MAM7). Values expressed as a ration between the number of Lactobacillus strains UFC versus *V. parahaemolyticus* UFC after 3 h of monolayers 1:1 co-infection. The figure represents the result of three full biological replicates, each time in technical triplicate. The figure shows values expressed as the mean ± standard deviation of three full biological replicates, each time in technical triplicate. *p < 0.001 (Student’s-Test)

MAM-7 is an outer membrane protein involved in host cell early adhesion in Gram negative pathogens including *V. parahaemolyticus* [6]. Thus, our objective was to determine if the expression of MAM-7 from RIMD in *L. rhamnosus* promotes bacterial adhesion to both human epithelial cell lines CaCo-2 and HEp-2 (Fig. 5a). As shown in Fig. 5a, MAM-7 did not promote higher adhesion in recombinant *L. rhamnosus* strains than the *L. rhamnosus* wild type. Furthermore, *L. rhamnosus* pSEC-MAM7 (pSEC-MAM7) showed significantly lesser adhesion than *L. rhamnosus* pCWA-MAM7 (pCWA-MAM7) and the *L. rhamnosus* wild type. In addition, all *L. rhamnosus* strains (recombinants and wild type strains) were significantly more adhesive than RIMD.

### Inhibition of *V. parahaemolyticus* from colonizing HEp-2 and CaCo-2 cell lines

Competition assay: Krachler et al. demonstrated that *E. coli* BL21 expressing MAM-7 inhibits *V. parahaemolyticus* attachment in HeLa cells [7, 9]. Thus, we expected that recombinant *L. rhamnosus* strains inhibit *V. parahaemolyticus* attachment in HEp-2 and/or CaCo-2 cell lines. Figure 5b shows that all *L. rhamnosus* strains efficiently competed with RIMD in HEp-2 cell line but *L. rhamnosus* wild type inhibition was higher than the recombinant strains. In CaCo-2, only *L. rhamnosus* wild type inhibited RIMD adhesion. Hence, *L. rhamnosus* wild type inhibited the adherence of *V. parahaemolyticus* to HEp-2 and Caco-2 cell lines more efficiently than recombinants strains.

**Fig. 6:**
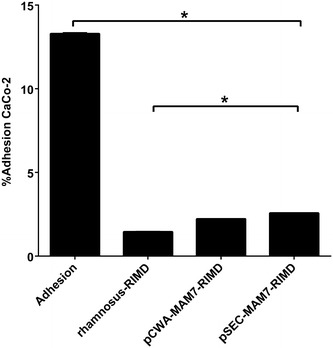
Competitive exclusion of *L. rhamnosus* to protect CaCo-2 cell line from *V. parahaemolyticus* colonization. The figure shows the results about the role of *L. rhamnosus* strains in the inhibition of *V. parahaemolyticus* from colonizing colon carcinoma cell line (CaCo-2) monolayers. The strains used were *V. parahaemolyticus* RIMD 2210633 (RIMD), *L. rhamnosus* wild type (*L. rhamnosus*), *L. rhamnosus* pCWA-MAM-7 (pCWA-MAM7) and *L. rhamnosus* pSEC-MAM-7 (pSEC-MAM7). Values are expressed as the adhesion percentage of *V. parahaemolyticus* after 3 h of *L. rhamnosus* wild type (*L. rhamnosus–* RIMD), pCWA-MAM-7 (pCWA-MAM7– RIMD) or pSEC-MAM-7 (pSEC-MAM7– RIMD) strains monolayer colonization. *V. parahaemolyticus* adhesion, without previous *Lactobacillus* treatment, is expressed as “Adhesion” *bar*. The figure shows values expressed as the mean ± standard deviation of three full biological replicates, each time in technical triplicate. *p < 0.001 (Student’s-Test)

Exclusion assay: To asses if *V. parahaemolyticus* could adhere to CaCo-2 already colonized with *L. rhamnosus* wild type or recombinant strains, we performed an exclusion assay in this cell line (Fig. 6). All *L. rhamnosus* strains significantly excluded RIMD from the CaCo-2 cell line. However, the *L. rhamnosus* wild type was significantly better than recombinant strains in excluding the pathogen. In fact, when RIMD was incubated with CaCo-2, a 16 % of adhesion was obtained. However, when we pre-colonized CaCo-2 with *L. rhamnosus* wild type, only 1 % of the adhered pathogen was recovered.

### Cytotoxic effect of *L. rhamnosus* expressing MAM-7

**Fig. 7:**
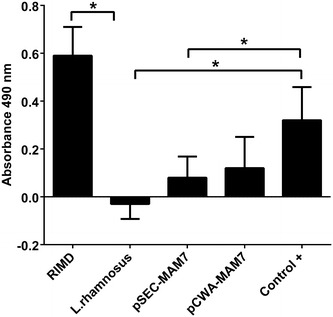
Cytotoxicity of *V. parahaemolyticus* and recombinants *L. rhamnosus* strains in CaCo-2 cell line. The figure shows LDH level release as absorbance at 490 nm of *V. parahaemolyticus* and *Lactobacillus* strains in colon carcinoma cell line (CaCo-2) monolayers. The strains used were *V. parahaemolyticus* RIMD 2210633 (RIMD), *L. rhamnosus* wild type (*L. rhamnosus*), *L. rhamnosus* pCWA-MAM-7 (pCWA-MAM7) and *L. rhamnosus* pSEC-MAM-7 (pSEC-MAM7). As a positive control (Control+), CaCo-2 were treated with Tritón  ×100. The figure represents the result of three full biological replicates, each time in technical triplicate. *p < 0.001 (Student’s-Test)

It has been reported that MAM-7 mediates *V. parahaemolyticus* TDH- independent cytotoxicity [6]. Thus, we wanted to know if *Lactobacillus* recombinant strains could generate cytotoxicity in CaCo-2 cell lines. Both pSEC-MAM7 and pCWA-MAM7 were significantly more cytotoxic than *L. rhamnosus* wild type (Fig. 7). However, cytotoxicity levels were significantly lower than the positive control. Our results suggest that the presence of MAM-7 in *L. rhamnosus* can induce cytotoxicity in the CaCo-2 cell line.

## Discussion

We evaluated the anti-adhesion capacity of *L. rhamnosus* recombinant strains against *V. parahaemolyticus* compared to the wild type, in HEp-2 and CaCo-2 epithelial cell lines.

Adhesion of recombinant *L. rhamnosus* strains was not better than that of *L. rhamnosus* wild type. In fact, in CaCo-2, an epithelial cell line that mimics epithelial cells of human colon, recombinant strains adhesion levels were significantly lower. *L. rhamnosus* are some of the most adhesive *Lactobacillus* species in CaCo-2 and HEp-2 cell lines [20, 21] and their adhesive capacity is mediated by numerous and varied surface proteins (such as S-layers proteins) and other structural molecules that form part of their surface architecture [22–24]. However, in Gram-negative bacteria, MAM-7 *mce* domains mediate attachment to host tissues by interaction with host fibronectin and membrane lipid phosphatidic acid (PA) [6]. Thus, bacteria that have the ability to bind to host fibronectin can be inhibited by the presence of MAM-7. Such is the case of the Gram-positive pathogen *Staphylococcus aureus*, which employs adhesins to attach and invade host cells through the interaction with fibronectin and can be inhibited by MAM-7 derived peptides [9, 25]. Recently, it was found that *L. rhamnosus* possesses a mucus-binding factor (MBF) that binds to mucin, laminin, collagen and fibronectin [26, 27]. Thus, the presence of attached or secreted MAM-7 in cell cultures could inhibited recombinant *L. rhamnosus* adhesion to epithelial cell lines and/or negatively interfere with intrinsic adhesion mechanisms of *L. rhamnosus.*

In our study, the presence of MAM-7 in *L. rhamnosus* induced cytotoxicity in CaCo-2 cell lines, suggesting that *V. parahaemolyticus* MAM-7 may trigger death in this adenocarcinoma cell line. In addition, Lim et al. demonstrated that *V. parahaemolyticus* MAM-7 is sufficient to disrupt the epithelial barrier and promote cell lysis. In HeLa as well as in the CaCo-2 cell line, MAM-7 triggered disruption of the epithelial barrier and a marked decreased in transepithelial electrical resistance (TER) [28].

*V. parahaemolyticus* MAM-7 recognizes host membrane PA lipids and can cluster on cell surface triggering the activation of the small GTPase RhoA [28]. In the same way, activation of Rho-associated serine/threonine kinase (ROCK) was observed downstream RhoA [28]. ROCK protein signalling can acts both a pro- and anti-apoptotic fashion depends of cell type, cell context and microenvironment [29]. ROCK family contains two members: ROCK1 and ROCK2 [30]. ROCK1 is related with cellular blebbing and blebbing is a conserved event of the apoptotic execution phase [29, 31]. Given that release of lactate dehydrogenase is related with either apoptosis or necrosis events, the level of LDH activity as a result of MAM-7 expression in *L. rhamnosus* could be related with apoptotic processes related with ROCK activation. Although there are a number of studies of pro-survival role of ROCK, ROCK proteins are essential for multiple aspects of both the intrinsic and extrinsic apoptotic processes [29]. The molecular mechanisms that modulate these pleiotropic roles are largely unknown. Thus, *Lactobacillus* expression of MAM-7 could have a role inducing apoptosis in CaCo-2 carcinoma cell line.

## Conclusion

*L. rhamnosus* can inhibit adhesion of *V. parahaemolyticus* in human epithelial cell lines. Expression of *V. parahaemolyticus* MAM-7 adhesin in *L. rhamnosus* decreases the intrinsic capacities of *L. rhamnosus* to inhibit *V. parahaemolyticus* in vitro.

## Methods

### Bacterial strains and culture conditions

*V. parahaemolyticus* (RIMD 2210633) serotype O3:K6 was obtained from Instituto de Salud Pública, (ISP, Chile). The strain was routinely grown in Luria–Bertani broth supplemented with 1,5 % NaCl (LBS) (Merck) and cultured with agitation at 37 ℃ for 12–24 h. *L. rhamnosus* LL1 was previously isolated from fermented dairy food, and was grown in de Man Rogosa and Sharpe broth (MRS, Merck) at 5–10 % CO_2_ (capnophilically) and at 37 ℃ without agitation for 48 h.

### *L. rhamnosus* recombinants strains

The MAM-7 gene was amplified from genomic DNA from *V. parahaemolyticus* using the primers MAM7-F (CCGATATCTCCGTATGTGCCTGATGTTAAGAGGA) and MAM7-R (CCAATGCATCAAGGGCTTAGGAATTGGCGTT) designed from *V. parahaemolyticus* reported sequence RIMD 2210633 (GenBank: NC_004603.1). PCR amplification was performed using 1.25 U of Dream Taq^®^ (Fermentas), 40 ng of genomic DNA from RIMD, and 65 ℃ of annealing temperature (30 cycles). The amplification product was cloned in pSEC and pCWA plasmids at *EcoR*V and *Nsi*I restriction sites to yield the plasmids pSEC-MAM7 and pCWA-MAM7. Resulting recombinant vectors were used to transform *L. rhamnosus* according to Serror et al. [32]. Presence of MAM-7 gene in *L. rhamnosus* strains was confirmed by PCR amplification and restriction endonuclease analyses. Expression of MAM-7 from each plasmid was assessed by RT-PCR using primers MAM_RT_-F (GCTCTACCTGACGACAACAAAGT) and MAM_RT_-R (TTCCTATCGGTGCGGTCTAC) (Fig. 1) and Western blot (Data not shown).

### Protein extraction

40 mL of MRS cultures of each recombinant *L. rhamnosus* and wild type strains was centrifuged at top speed for 2 min and washed once with PBS 1X (Gibco). Bacterial pellets were resuspended in digestion buffer (50 mM Tris–HCL pH 8,0; 5 mM MgCl2; 50 mM EDTA; 10 mg/mL lysozyme) and incubated by 3 h at 37 ℃. Glass pearls were then added and lysates were vortexed for 120 s. Then, the lysates were centrifuged at 7000x*g* for 15 min. Supernatants were recovered and sonicated in ice three times for 10 s each. Proteins were precipitated with 10 % trichloroacetic acid (TCA, Merck) for 12 h and then centrifuged at top speed at 4 ℃ for 20 min. Supernatants were discarded, and pellets were washed once with cold acetone and centrifuged at top speed at 4 ℃ for 5 min. Obtained pellets were resuspended with buffer (100 mM Tris–HCl pH 8,0) and stored at −20 ℃ until their use.

### Sodium dodecyl sulfate polyacrylamide gel electrophoresis (SDS-PAGE)

To evaluate the presence of MAM-7 protein, a SDS-PAGE was performed. A polyacrylamide gel (with 10 % acrylamide in separating gel) was prepared according to standard protocol. 50 μg of each protein sample was loaded and gel was run in a Mini-PROTEAN chamber at 100 V for 2 h (BioRad) in pH 8,3 Tris-glicine-SDS buffer. The gel was stained with silver using the Pierce Silver Stain Kit (Thermo Fisher Scientific) according the instruction of manufacturer. Gel electrophoresis bands were analyzed using freeware 1D gel electrophoresis image analysis software GelAnalyzer (http://www.gelanalyzer.com/index.html).

### Fluorescent immunodetection assay

To asses if recombinant *L. rhamnosus* strains expressed MAM-7, bacterial samples were placed onto coverslips previously treated with poly-l-lysine (Sigma) for 20 min at room temperature. Each coverslip with the sample was dried at 37 ℃ and fixed with 3 % paraformaldehyde (PFA, Merck) for 15 min at room temperature. Then, coverslips were put on 24-well cell culture plate (TrueLine) and washed three times with PBS 1 × (Gibco). Samples were then blocked using 1 % albumin bovine serum (BSA, Calbiochem^®^) for 1 h at room temperature in humidity chamber. Each sample was incubated with 50 μL of 1:50 anti-*Vibrio* primary antibody (BacTrace anti-vibrio species antibody, KPL) for 12 h at 4 ℃. Once time had passed, each sample was washed with PBS 1 × (Gibco) and then, incubated with 50 μL of 1:500 goat anti-rabbit secondary antibody alexa fluor^®^ 488 conjugate (molecular probes) in darkness and humidity chamber for 2 h. After that, samples were washed three times with PBS (Gibco) and coversilps were mounted over microscope slides with Dako fluorescent mounting medium (Fluoromont-g Emsdiasum) for 1 h in darkness. Image adquisition was performed in Leica TCS SP8, DMI6000 CS (Leica Microsystems). Adquisition was performed with argon laser, HC PL APO CS2 63x/1.40 oil objective, and optic slices were set at 0.895 μm.

### Growth curves

To assess if MAM-7 expression alters the growth of *L. rhamnosus* recombinants strains, *Lactobacillus* strains were cultivated in MRS broth (wild type), or MRS supplemented with chloramphenicol (recombinants strains) at 5–10 % CO_2_ at 37 ℃. Then, an aliquot of each culture was aseptically obtained at 0, 2, 17, 26 and 45 h and serially diluted in MRS. The bacterial concentration was determined according the number of CFU/mL of MRS. The assay was performed in triplicate.

### Biofilm formation

To assess if MAM-7 expression alters intrinsic properties of *Lactobacillus* as biofilm formation, wild type and recombinant *Lactobacillus* strains were cultivated in MRS broth (wild type), or MRS supplemented with chloramphenicol (recombinants strains) at 5–10 % CO_2_ at 37 ℃ for 24 h. After the time, optical density at 600 nm (OD_600nm_) was measured. A volume of 100 μL of bacterial cultures adjusted at OD_600nm_ 0, 2 were used to inoculate sterile 96-well plates (TrueLine) (10^8^ cells/well) and grown at 5–10 % CO_2_ at 37 ℃ for 5 days. Sterile MRS broth was used as a control.

### Crystal violet staining

To assess biofilm formation, crystal violet staining protocol was used. Cells suspensions were removed from test wells before washing three times with PBS. 100 μL of methanol (Winkler) was added to each test well and was incubated for 15 min at room temperature. After this treatment, methanol was discarded before drying. 100 μL of 2 % (w/v) crystal violet solution was added and wells were further incubated at room temperature for 5 min. After this incubation, the non-bound dye was removed and wells were washed with H_2_O. Bound crystal violet was dissolved using 33 % (v/v) of glacial acetic acid (Winkler). The resulting dissolved dye was measured at a wavelength of 590 nm using NanoQuant Infinite M200 PRO (TECAN).

### Adhesion assay

To assess bacterial adhesion to epithelial cells, both HEp-2 and CaCo-2 cell lines were grown in 96-well plates (TrueLine) (10^5^ cells/well) at 37 ℃ with 5 % CO_2_/95 % air atmosphere in DMEM High Glucose (HyClone). *V. parahaemolyticus* RIMD were grown overnight in LBS, while *L. rhamnosus* wild type and recombinant strains were grown overnight in a capnophilic environment in MRS broth (wild type), or MRS supplemented with chloramphenicol (recombinants strains) at 37 ℃. Bacterial cultures were harvested by centrifugation prior to colonisation of the cellular monolayers (approx. 10^8^ UFC/mL of each strain). After 3 h of co-incubation at 37 ℃ in 5 % CO2 to allow bacteria to adhere to eukaryotic cells, monolayers were washed twice with phosphate-buffered saline (PBS, Gibco). Cells were then lysed with Tritón X-100 (1 %, in PBS), and the quantity of bacteria was determined. Quantitative adhesion assay values were calculated as follows:

% Adhesion = 100 × [(UFC mL^−1^ at 3 h post-colonization)/(UFC mL^−1^ added)]. The output is the result of three independent assays and each experiment was made in triplicate.

### Competition assay

In this assay, *L. rhamnosus* strains (10^8^ CFU/mL) and *V. parahaemolyticus* (10^8^ CFU/mL) were added simultaneously to CaCo-2 and HEp-2 cell lines. The strains were then co-incubated for 3 h at 37 ℃ with 5 % CO_2_/95 % air atmosphere. After the incubation period, non-adhered bacteria were removed by washing with PBS, and adhered bacterial cells were recovered by lysis of eukaryotic cells with Tritón X-100 (1 %, in PBS). Bacterial count was expressed as: Ratio = [(UCF mL^−1^*L. rhamnosus* strain)/(UFC mL^−1^*V. parahaemolyticus*)] at 3 h. The output is the result of three independent assays and each experiment was made in triplicate.

### Exclusion assay

For exclusion assays, approximately 10^8^ CFU/mL of *Lactobacillus* strains (wild type or recombinant strains) were added to confluent CaCo-2 monolayers in 96-well plates, in presence of 5 % CO_2_/95 % air atmosphere at 37 ℃. After 3 h of co-incubation to allow bacteria adhere to eukaryotic cells, monolayers were washed twice with PBS (Gibco) to remove non-adhered bacteria. Then, 10^8^ CFU/mL of *V. parahaemolyticus* was added to CaCo-2 already colonized by *L. rhamnosus* strains, which allowed further incubation of 3 h in presence of 5 % CO_2_/95 % air atmosphere at 37 ℃. After the co-incubation period, non-adhered bacteria were removed by washing with PBS, and bacteria were recovered by lysis of eukaryotic cells with Tritón X-100 (1 %, in PBS). Bacterial counts were expressed as percentage of adhered *V. parahaemolyticus,* as mentioned earlier. The output is the result of three independent assays and each experiment was made in triplicate.

### LDH cytotoxicity assay

To assess if the expression of MAM-7 in *L. rhamnosus* can induce cytotoxicity in CaCo-2 cells, an LDH-Cytotxicity assay was performed. *V. parahaemolyticus* RIMD were grown overnight in LBS, while *L. rhamnosus* wild type and recombinant strains were grown overnight in a capnophilic environment in MRS broth (wild type), or MRS supplemented with chloramphenicol (recombinants strains) at 37 ℃. Bacterial cultures were harvested by centrifugation prior to colonisation of the cellular monolayers (approx. 10^8^ UFC/mL of each strain). After 4 h supernatants from CaCo-2 cells colonized by *V. parahaemolyticus* or *L. rhamnosus* strains was assayed using the Cyto Tox^®^ NonRadiactive cytotoxicity assay (Promega), which measures the extracellular release of LDH into media by dead cells, according manufacturer’s instructions. As a positive control, CaCo-2 were treated with Tritón X-100 for 45 min.

The absorbance values of treated cells were expressed at 490 nm after correcting for background from media without cells. The output is the result of three independent assays, and each experiment was carried out in triplicate.

### Statistics

Results are expressed as average ± SD of an individual experiment performed in triplicate. P values were calculated according to Student’s t test, and values p < 0.05 or p < 0.01 were considered statistically significant.

## Abbreviations

RL: recombinant *Lactobacillus* strains
AAT: anti-adhesion therapy
GRAS: generally recognized as safe
*V. parahaemolyticus*: *Vibrio parahaemolyticus*
*L. plantarum*: *Lactobacillus plantarum*
*L. brevis*: *Lactobacillus brevis*
*L. rhamnosus*: *Lactobacillus rhamnosus*
RIMD: *V. parahaemolyticus* RIMD 2210633 serotype O3:K6
pSEC-MAM7: pSEC plasmid containing *V. parahaemolyticus* MAM-7 gene
pCWA-MAM7: pCWA plasmid containing *V. parahaemolyticus* MAM-7 gene
